# MR urogram findings and diffusion restriction in the renal papilla and calyx in papillary necrosis—a new finding: preliminary report

**DOI:** 10.1259/bjrcr.20150476

**Published:** 2017-02-07

**Authors:** Denver Steven Pinto, Arun George, Ravi V Hoisala

**Affiliations:** Department of Radiodiagnosis, St Johns Medical College, Koramangala, Bangalore, India

## Abstract

Renal papillary necrosis is a clinicopathological entity where any or all of the papillae undergo selective necrosis, which can be demonstrated either radiologically or histologically. The most important causes are diabetes, pyelonephritis, obstructive uropathy, tuberculosis, analgesic abuse or overuse, sickle cell disease and renal vein thrombosis. Although this condition was first described in the 19th century the clinical diagnosis of this condition remains a problem to this day. Uncomplicated papillary necrosis may initially remain occult to imaging by ultrasound and non-contrast CT, but may later be complicated by obstructive uropathy. A few studies have described renal papillary necrosis on CT urogram. In this case series, the authors describe the finding of calyceal filling defect with diffusion restriction in the calyx and the tip of the renal pyramid on MR urogram, along with other findings that are classically seen on intravenous urogram or CT urogram. To the best of our knowledge, the finding of diffusion restriction at the tip of the renal pyramid has not been described before. Further, literature review showed only a single study describing the classical findings of papillary necrosis on an MR urogram. The early diagnosis of papillary necrosis on MR imaging equips the radiologist to suggest short-term clinical and radiological follow-up to check for the development of hydronephrosis. Additionally, such risk stratification may enable early ureteric stenting to prevent the development of obstructive uropathy.

Renal papillary necrosis is a clinicopathological entity where any or all of the papillae undergo selective necrosis that can be demonstrated either radiologically or histologically.^1^ Papillary necrosis has been described to be common in patients with diabetes.^2,3^ With the increasing number of patients with diabetes, who are approaching epidemic proportions, this condition has the potential to cause significant renal morbidity. The other causes of papillary necrosis are pyelonephritis, obstructive uropathy, tuberculosis, analgesic abuse or overuse, sickle cell disease and renal vein thrombosis. A few studies describing the findings of papillary necrosis on MR urogram exist. Along with the description of findings of papillary necrosis seen on intravenous urogram and CT urogram, in this case series the authors report the finding of diffusion restriction at the tip of the renal pyramid in renal papillary necrosis. The early diagnosis of this condition has the potential to alter management by either starting early treatment or withdrawing the inciting stimulus.^4^ The patients may be offered early stenting or percutaneous nephrostomy if the diagnosis of papillary necrosis can be established.

## History, clinical examination and imaging findings with follow-up

Case1: Reported here is a 41-year-old male patient who had previously undergone pyeloplasty for right pelviureteric junction obstruction. The patient was diabetic since 10 years and had developed chronic kidney disease 5 years ago. The patient presented with fever with chills, burning micturition and right flank pain.

On examination the patient was febrile. Urine examination done just before the MR urogram showed 32 white blood cells/hpf and 1 bacteria/hpf. The creatinine level of the patient was 3.9 mg dl^–1^, which prevented the administration of contrast.

**Figure 1.**
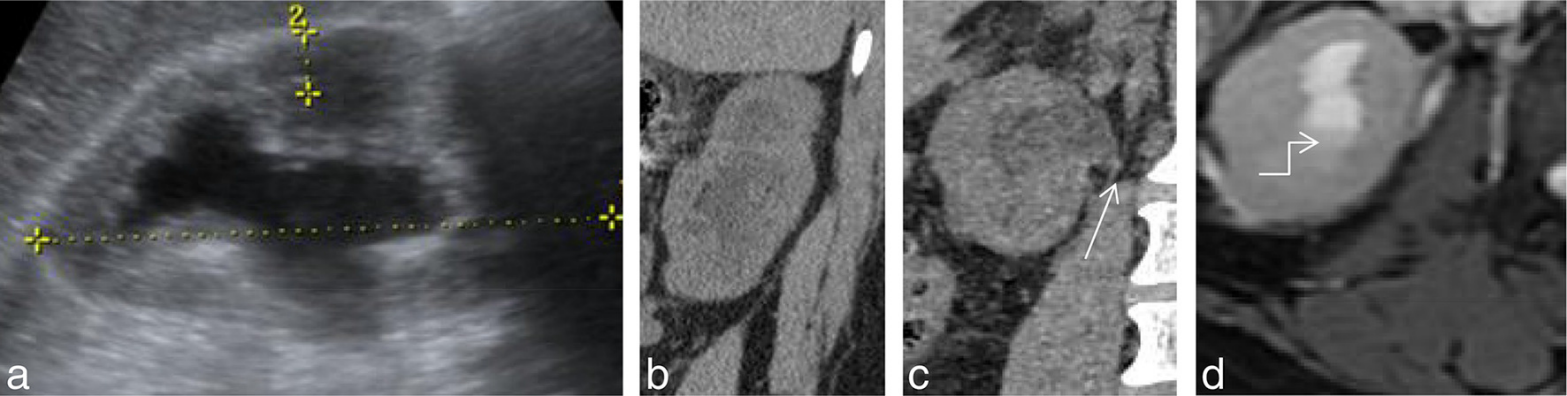
(a–d) Ultrasound image showing hydronephrosis (a) Non-contrast CT shows hydronephrosis (b) with sudden tapering of the renal pelvis (white arrow) at the pelviureteric junction. (c,d) Axial *T*_2_ weighted image showing perinephric fat stranding with debris (bent arrow) within the calyces.

CT imaging was performed, which showed perinephric fat stranding and hydronephrosis with sudden tapering of the right ureter at the pelviureteric junction (Figure 1).

MRI was performed to image the ureter and to diagnose the cause of obstruction. Clinical suspicion was of a post-pyeloplasty stricture. The patient’s high creatinine level prevented the administration of iodinated contrast.

**Figure 2.**
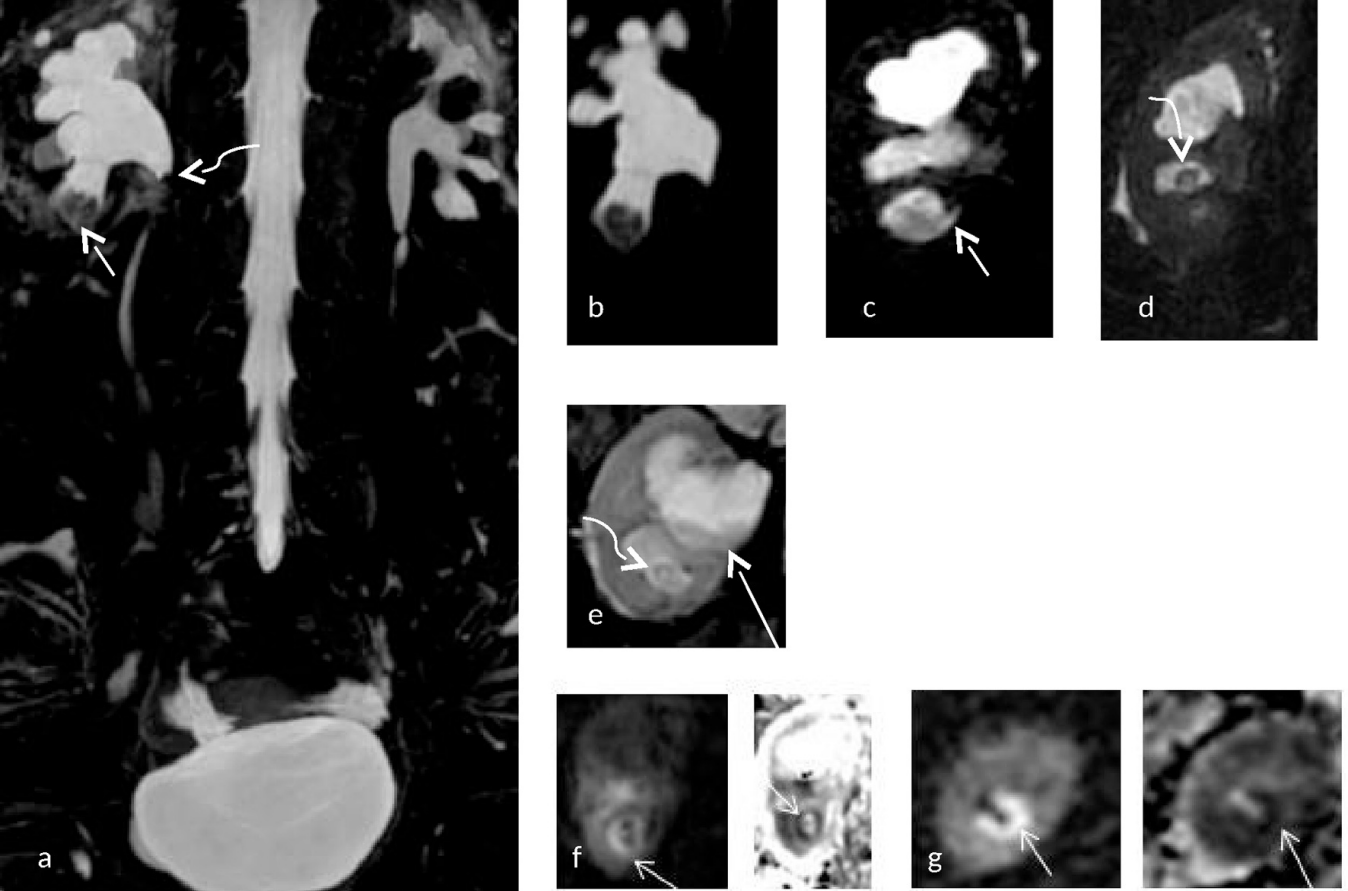
(a–g) (Same patient as in Figure 1) (a) Coronal maximal intensity projection of a heavily *T*_2_ weighted image showing a filling defect in the interpolar and lower polar calyx (short white arrow) with sudden tapering at the pelviureteric junction (curved arrow) on the right side. Mild hydronephrosis with narrowing at the pelviureteric junction is also noted on the left side. (b) Shows a magnified image with a filling defect in the lower polar calyx on the right side. (c) Shows a cleft (small white arrow) at the lower pole calyx. (d,e) Show filling defect in the interpolar calyx (curved white arrow) with debris (long white arrow) within the calyx. (f,g) Show diffusion restriction (small white arrow) at the tip of the medullary pyramids on the right side with their corresponding apparent diffusion coefficient maps [b = 0,800 s mm^–2^, TR (Relaxation time)5000 ms, TE(Echo time) 95 ms, ET(Echo train) = 1]. Apparent diffusion coefficient value measured at the lower pole calyx of the right kidney was 1.004× 10^–3^ mm^2^ s^–1^.

MRI showed hydronephrosis of the right kidney with sudden tapering at the level of the pelviureteric junction with diffusion restriction at the tip of the renal pyramid, with widening of the fornices, clubbing of the calyces and filling defects in the lower pole and interpolar calyx. Additionally, cleft was noted at the lower pole. These imaging findings were suggestive of a right pelviureteric junction stricture with papillary necrosis (Figure 2).

However,there was no growth of bacteria on urine culture for this patient. There was growth of Candida species other than *Candida albicans* on fungal culture. Nevertheless, the patient was started on broad-spectrum antibiotics with systemic antifungals with resultant resolution of fever and flank pain with the urinary white blood cell count returning to normal. Post treatment urine culture was normal.

Case 2: Reported here is a 48-year-old female patient who is a known case of diabetes with chronic kidney disease. She previously had bilateral emphysematous pyelonephritis, which resolved with treatment. Stenting was done for the patient at that time, which was removed with resolution of patient symptoms and normalization of routine urine analysis and culture. In the current episode the patient presented with right flank pain and fever. Serum creatinine level of the patient was 6.1 mg dl^–1^.

MRI was requested in view of bilateral hydronephrosis on ultrasound imaging with the clinical differential diagnosis of pyelonephritis and ureteric calculus.

**Figure 3.**
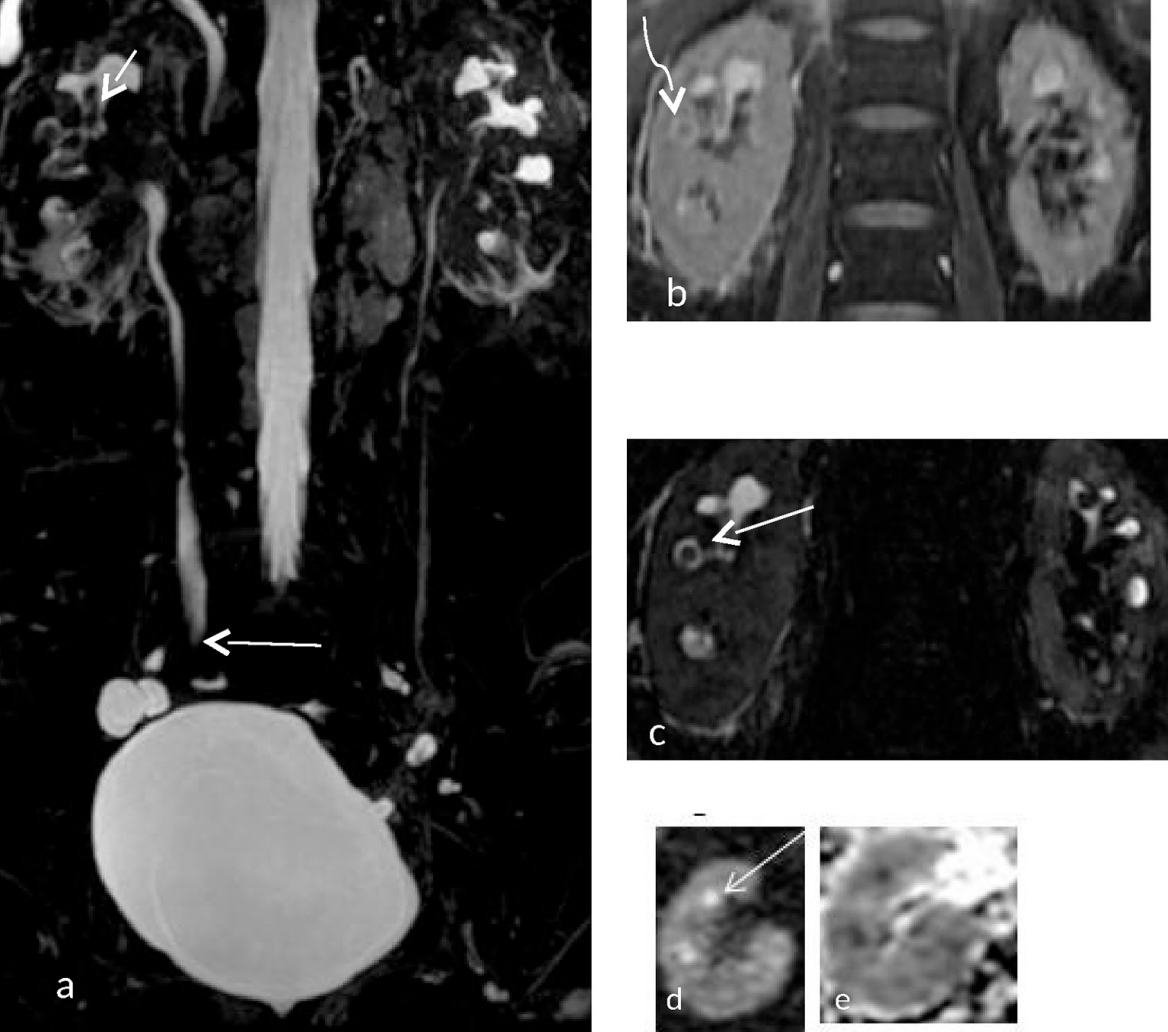
(a) Coronal maximum intensity projection of a 3D MR cholangiopancreatography sequence showing filling defects within the calyceal system (short straight white arrow) with sudden tapering of the right ureter at mid-ureteric level (Long straight white arrow). (b) Coronal 2 D fast spin echo image showing perinephric fat stranding around the right kidney with a filling defect (curved arrow) at the right interpolar calyx. (c) Shows a single section of a 3D MR cholangiopancreatography image showing *T*_2_ hyperintense urine around the interpolar calyx on the right side suggestive of a rim/ring sign (straight arrow). (d) Shows a diffusion-weighted image showing diffusion restriction (straight arrow) at the calyx and tip of the renal pyramid. The corresponding apparent diffusion coefficient image is provided in (e). [b = 0, 800 s mm^–2^, TR 4500 ms, TE 90 ms ET = 1]. Apparent diffusion coefficient value derived was 0.989×10^–3^ mm^2^ s^–1^.

**Figure 4.**
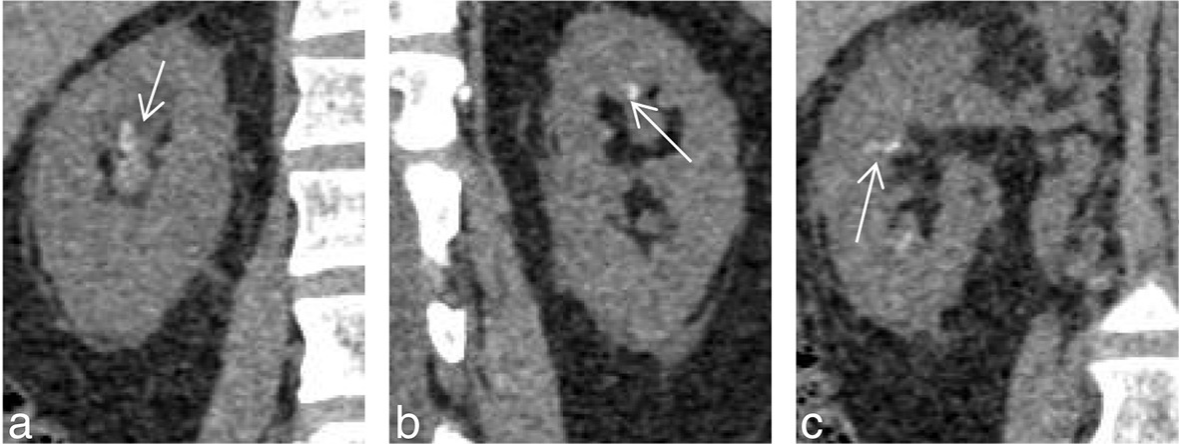
(a–c) (Same patient as in Figure 3) Coronal reformats of CT images showing papillary calcification in both kidneys (arrows).

MRI showed mild bilateral hydronepehrosis and bilateral perinephric fat stranding, which was more on the right side. Multiple filling defects were noted within the pelvicalyceal system on both sides, with diffusion restriction noted at the tips of two of the renal pyramids on the right side. A ring sign was seen in the calyces on the right side (Figure 3). Complimentary non-contrast CT was performed following the MRI, to definitively rule out a ureteric calculus. This CT showed papillary calcifications on both sides (Figure 4).

Urine examination of the patient showed 10 white blood cells/hpf and culture showed growth of Klebsiella. Right-sided ureteric stenting was performed. The patient was treated with antibiotics following which her symptoms reduced and urine routine and culture returned to normal.

**Figure 5.**
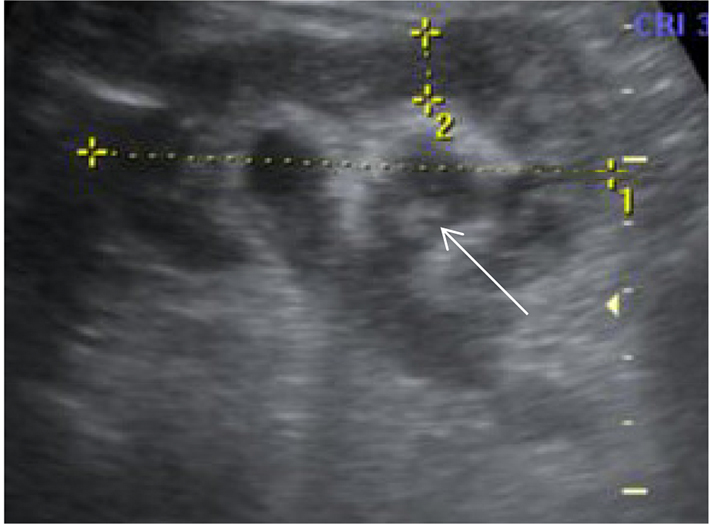
Ultrasound image showing hydronephrosis with a dilated pelvicalyceal system. Hyperechoic material is noted within the interpolar calyx (long white arrow).

Case 3: Reported here is a 66-year-old non-hypertensive, non-diabetic female patient, known to have chronic renal failure who presented with progressively increasing flank pain. Initial evaluation by ultrasound imaging showed left-sided hydronephrosis (Figure 5). The creatinine level of this patient was 4.5 mg dl^–1^.

**Figure 6.**
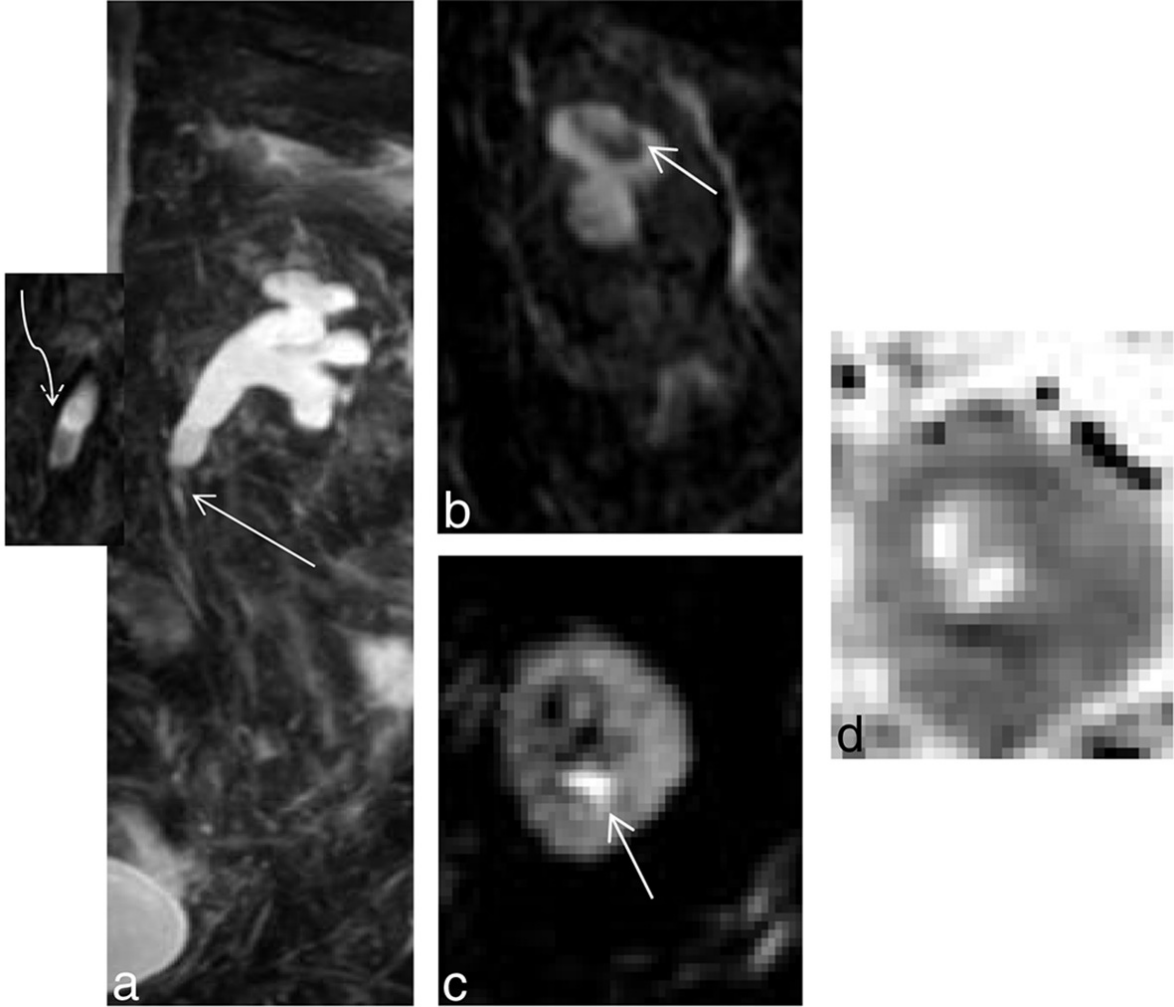
(a–c) (Same patient as in Figure 5) (a) -Showing sudden tapering (straight arrow) with filling defect (curved arrow at insert) in the upper ureter causing hydroureteronephrosis. (b) Showing filling defect(straight arrow) in the upper pole calyx. (c) Shows diffusion restriction in the upper polar calyx and the tip of the pyramid (straight arrow). (d) shows the apparent diffusion coefficient map which shows hypointensity corresponding to the region of diffusion restriction. [b = 0, 800 s mm^–2^, TR 4500 ms, TE 90 ms ET = 1]. Apparent diffusion coefficient value derived was 1.090×10^–3^ mm^2^ s^–1^.

MRI was requested to rule out pyelonephritis. MRI showed filling defect in the proximal ureter causing hydroureteronephrosis. Further, coronal slice of the 3D MR cholangiopancreatography image showed filling defect in the upper polar calyx and diffusion-weighted images showed diffusion restriction in the calyx and renal pyramid (Figure 6).

The patient underwent ureteric stenting under antibiotic coverage, where a brownish black irregular slough was removed. This was followed by relief of symptoms. The stent was removed after 3 months and the patient had an uneventful course at 6 month follow-up.

Case 4: Reported here is a 47-year-old male who underwent radical cystoprostatectomy with bilateral ureteric reimplantation into the ileum for bladder carcinoma. The patient subsequently developed increased creatinine and acute renal failure with a creatinine level of 6.6 mg dl^–1^. Laboratory evaluation showed increased white blood cell count and the presence of bacteria in urine routine examination. MRI was requested to establish the diagnosis of pyelonephritis and to rule out the recurrence of bladder carcinoma.

**Figure 7.**
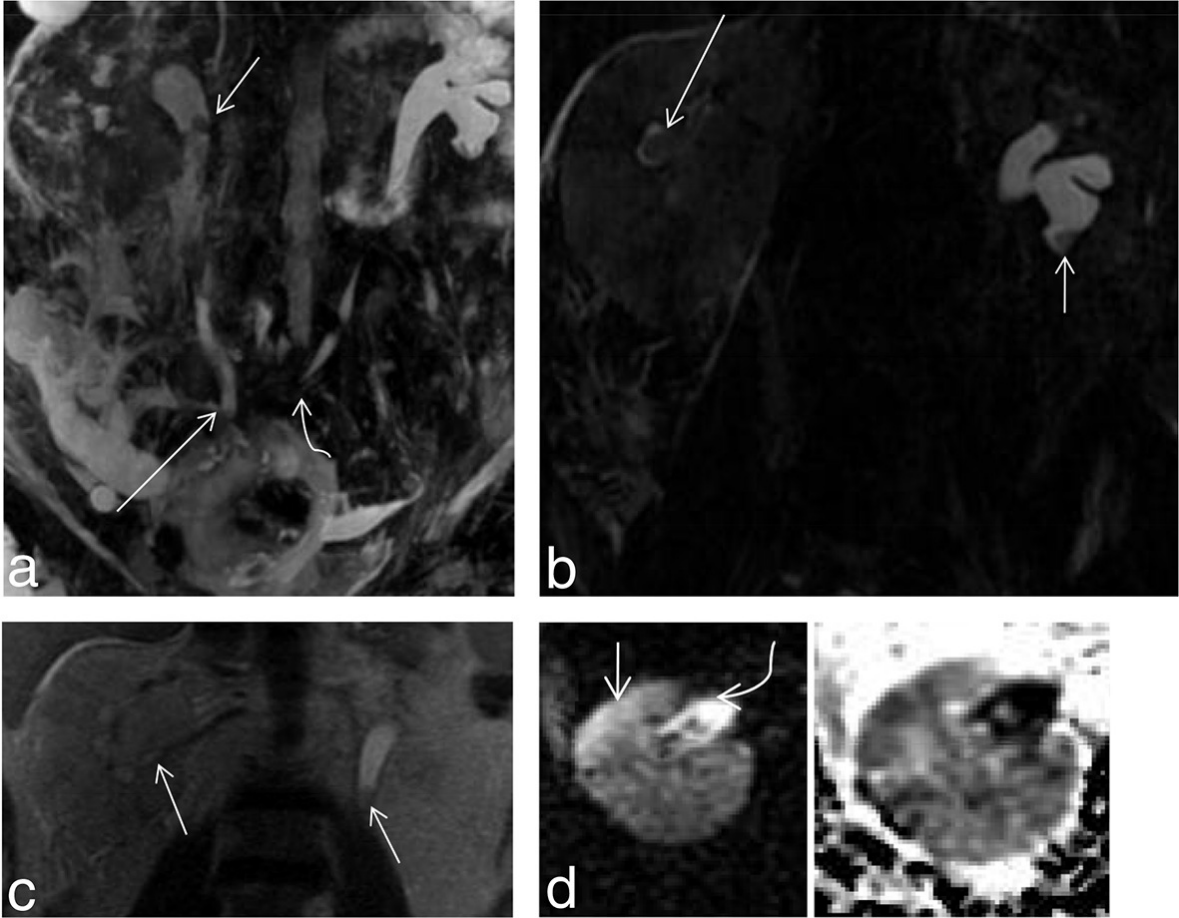
(a) Shows a coronal maximum intensity projection of a 3D MR cholangiopancreatography sequence showing a filling defect in the upper ureter (short straight arrow) and non-visualized right lower pole calyx suggesting a phantom calyx sign. Also noted in this image is the termination of the right ureter in the ileum (long straight arrow) and a medially deviated left ureter (curved arrow). (b) Shows a filling defect in the lower pole calyx (short straight arrow) of left kidney with a ring sign (long straight arrow) in the right interpolar calyx. (c) Shows a coronal *T*_2_ weighted image of a bulky right kidney, with a filling defect in the renal pelvis, calyces and proximal ureter (straight arrows). (d) Shows a diffusion-weighted image with corresponding apparent diffusion coefficient map showing a focus of parenchymal diffusion restriction (short straight arrow) with diffusion restriction in the renal pelvis (curved arrow) suggesting pyelonephritis with pyonephrosis. The corresponding ADC hypointensity is noted. Thus a diagnosis of pyelonephritis with papillary necrosis and pyonephrosis was made.

**Figure 8.**
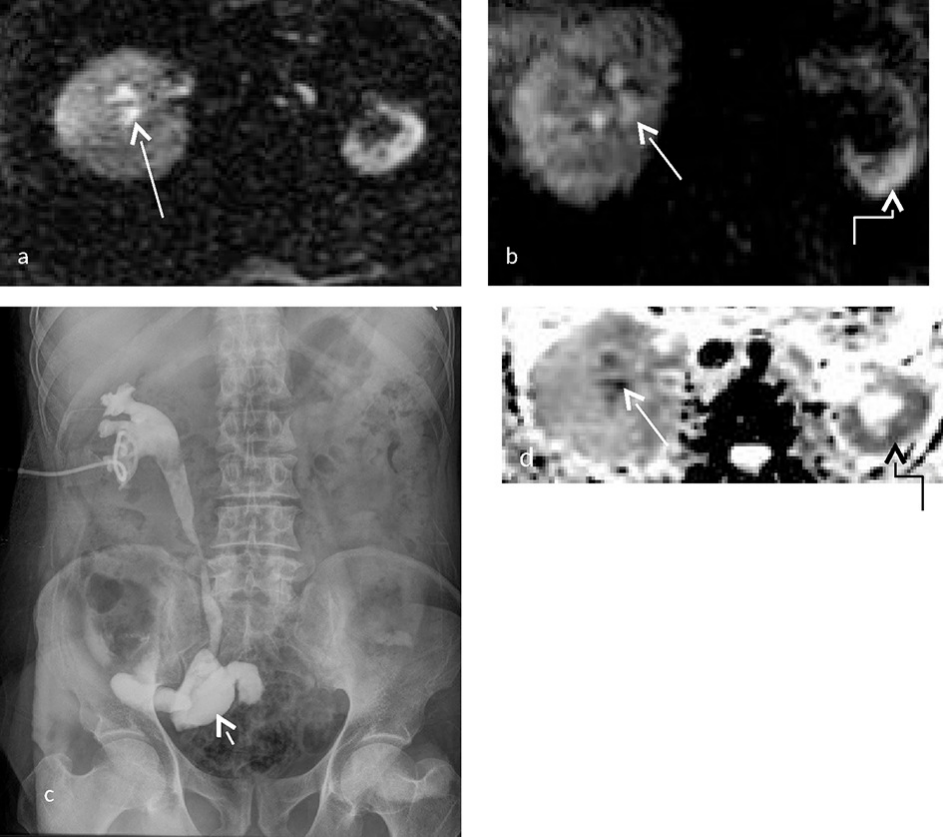
(a–d) (Same patient as in Figure 7) (a,b) Show diffusion restriction within the calyces of the right kidney with no evidence of calyceal diffusion restriction in the left kidney. However, subtle parenchymal diffusion restriction is noted in the left kidney. Apparent diffusion coefficient (ADC) images are provided in (d) showing the corresponding ADC hypointensity [b = 0,800 s mm^–2^, TR 4500 ms, TE 90 ms ET = 1]. ADC value at the calyces of the right kidney was 0.859×10^–3^ mm^2^ s^–1^. (c) Shows a percutaneous nephrostogram performed through a percutaneous nephrostomy tube with narrowing of the mid-ureteric segment with free flow of gastrograffin into the ileum (arrow). Irregular calyces are noted in the right kidney.

Evaluation by MRI showed a medially deviated left ureter with a filling defect in the left lower pole calyx. Further, a bulky right kidney with areas of parenchymal diffusion restriction surrounded by free fluid with filling defects in the right ureter, calyces and the renal pelvis suggested a diagnosis of right-sided pyelonephritis with bilateral papillary necrosis (Figure 7). Further evidence of diffusion restriction was seen at the calyces and the tips of the renal pyramids (Figure 8).

The medially deviated left ureter probably represents its post surgical course, ultimately terminating in its ureteric implantation. The filling defect in the left lower pole represents old papillary necrosis, either secondary to obstruction or due to an older event of pyelonephritis. This slough poses a risk of causing obstructive uropathy and hence left-sided percutaneous nephrostomy was done as a preventive measure.

In view of ureteric reimplantation into the ileum, a right percutaneous nephrostomy was done with removal of slough. After the procedure, a nephrostogram showed free flow of contrast through the right pelvicalyceal system into the ileum (Figure 8).

The urine culture of the patient showed growth of *Proteus vulgaris*. The patient improved with antibiotics and was put on twice weekly follow-up. This was followed up by a percutaneous nephrostomy on the left side.

Case 5: Reported here is a 64-year-old male patient, a known case of diabetes since 18 years, who presented with fever and burning micturition. The patient had an elevated creatinine level of 3.5 mg dl^–1^ and presented with back pain and fever raising a suspicion of pyelonephritis. Ultrasound imaging showed bilateral hydroureteronephrosis. Laboratory investigation revealed increased white blood cell count.

**Figure 9.**
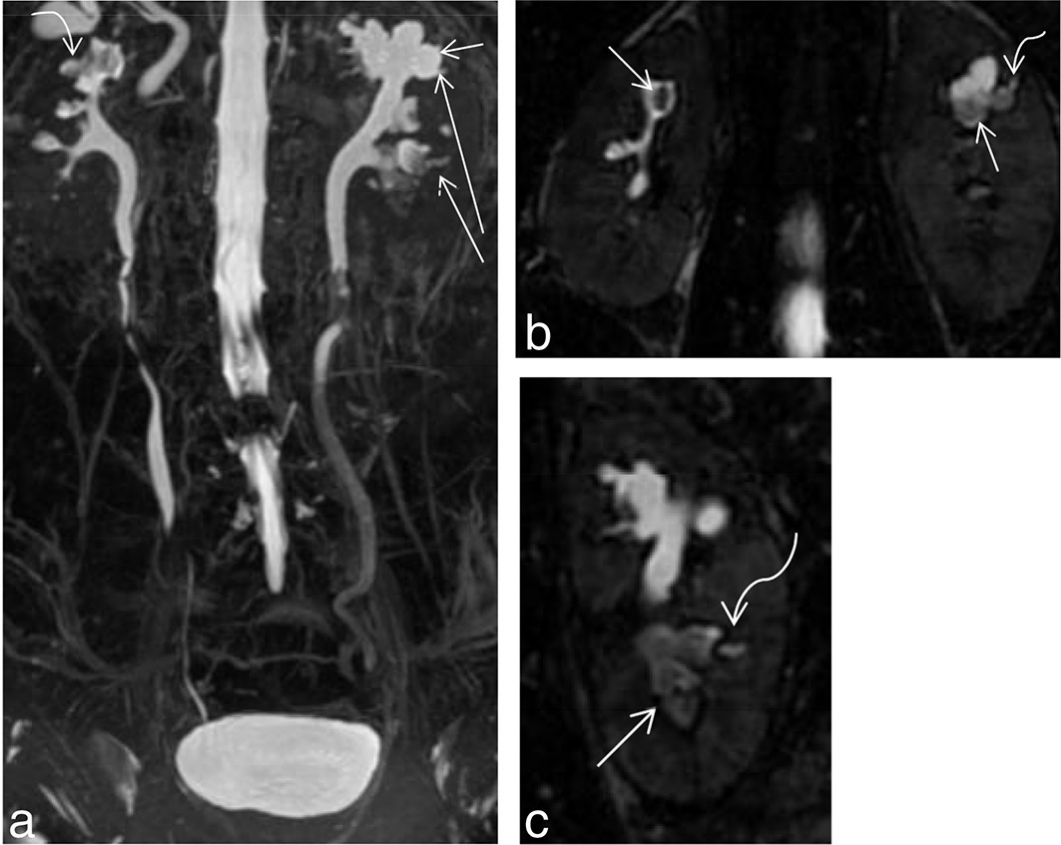
(a–c) (a) shows a 3D MR cholangiopancreatography image of the patient with bilateral papillary necrosis with blunting of the calyces (short arrow), formation of clefts at the renal pyramid (long arrow) and filling defects within the calyces (curved arrow). Coronal sections through the kidneys in (b) show a cleft (curved arrow) at the left upper polar calyx with filling defects in the upper pole calyces of both kidneys (small white arrows). (c) Shows a lower pole calyx filled with slough also known as phantom calyx (straight arrow). A cleft is also noted (curved arrow).

**Figure 10.**
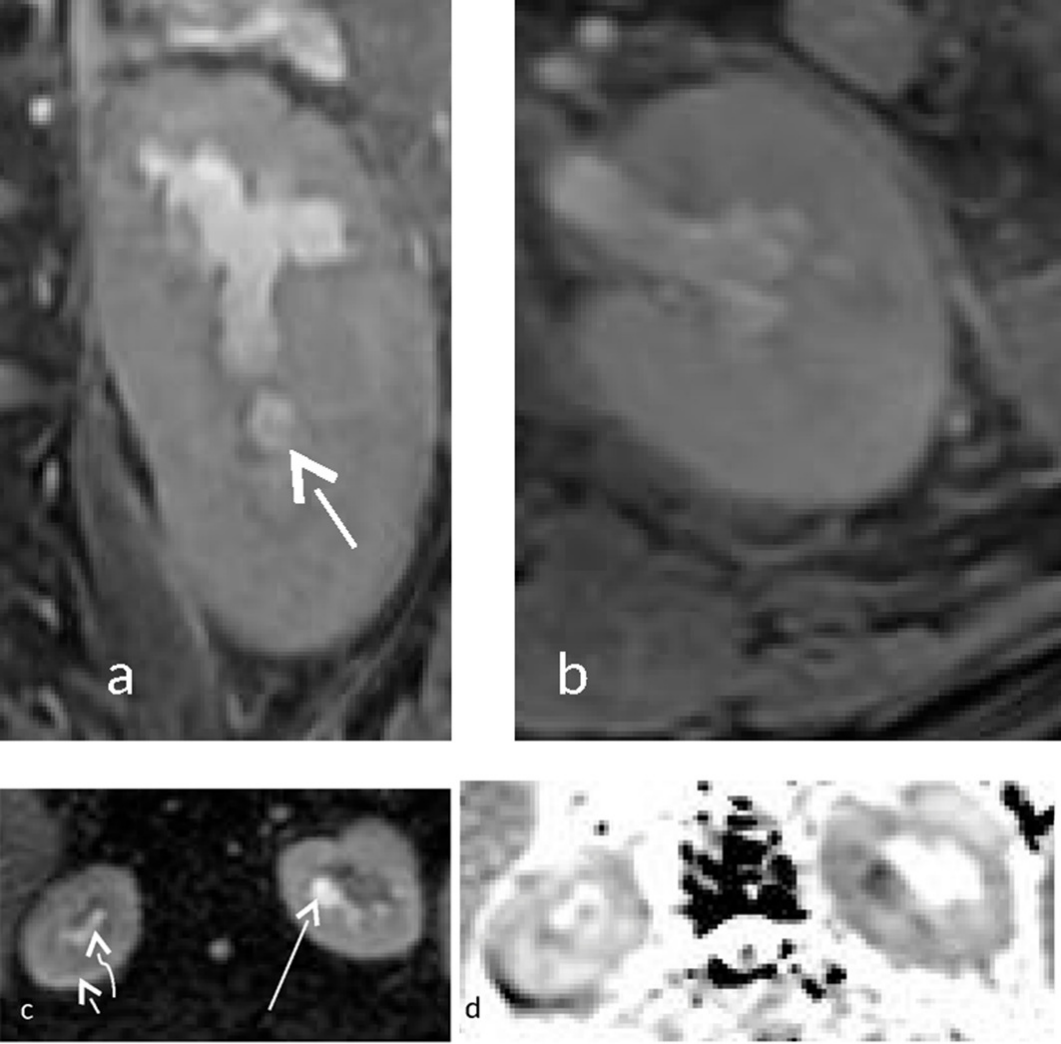
(a–d) (Same patient as in Figure 9) (a, b) Coronal and axial *T*_2_ weighted images of the left kidney shows irregular calyces with blunting, with intermediate signal intensity in the left lower pole (small arrow). (c) Shows true diffusion restriction of a thin rim of renal parenchyma on the right side (short white arrow) and true diffusion restriction of the calyces on the left side (long white arrow). Subtle diffusion restriction was noted in the calyces of the right kidney (curved arrow). Corresponding apparent diffusion coefficient hypointensity was noted in both kidneys [b = 0, 800 s mm^–2^, TR 4500 ms, TE 90 ms ET = 1]. Apparent diffusion coefficient value derived at the calyces of the left kidney was 0.989× 10^–3^ mm^2^ s^–1^.

MRI was requested to elucidate the cause of the bilateral hydronephrosis. Evaluation by MRI additionally showed filling defects in the calyces of both kidneys (Figure 9). Further, the right kidney showed a thin rim of parenchymal diffusion restriction with diffusion restriction of the calyces and the tip of the medullary pyramids in both kidneys (Figure 10). This suggested a possibility of early right-sided pyelonephritis with a diagnosis of bilateral papillary necrosis. Other features of papillary necrosis such as filling defects, clefts and phantom calyces were seen. Urine culture demonstrated the growth of *Escherichia coli* (Figure 9).

The patient underwent bilateral ureteric stenting, followed by a course of antibiotics. This resulted in improvement of patient symptoms with return of routine urine examination and urine culture to normal.

Thus diffusion restriction at the calyx and tip of the renal pyramid with filling defects within the pelvicalyceal system was seen in all five cases of papillary necrosis. A cleft in the region of the tip of the renal pyramid was seen in two of the five patients. This led us to conclude that diffusion restriction at the calyx and tip of the renal pyramid with filling defects within the pelvicalyceal system is an important finding in papillary necrosis.

Also to be noted in this series is that all patients had elevated creatinine levels, which prevented the use of intravenous urography (conventional) or contrast-enhanced CT or MR urography. MRI can provide valuable information by imaging the urine-filled ureter. Thus, at our institution, in this subset of patients, imaging with MRI is preferred. Further, in contrast to CT, MRI does not deliver any radiation dose to the patient. This becomes important since many patients with diabetes and patients with renal failure undergo multiple imaging examinations.

## Discussion

In this case review the authors discuss the findings of papillary necrosis on MR urogram. The patients showed calyceal filling defects with or without diffusion restriction, filling defects with clubbing of calyx, ring shadow, blunt tipped calyx, phantom calyx and clefts extending from the calyces to the pyramids. These findings of papillary necrosis have classically been described on intravenous urography and later on CT urography.^5^

Jung DC et al described the findings of papillary necrosis on CT urogram where they divided the disease process of papillary necrosis into four phases:

a) Early ischaemic changes in the medullary pyramid; b) clefts from the calyceal fornices to the medullary pyramid; c) sloughing of the papilla and d) the healing phase. Renal papillary necrosis is the consequence of an ischaemia in the renal papillae. Infections such as pyelonephritis causing inflammation of the interstitium may lead to compression of the medullary vasculature and predispose the renal papillae to ischaemic change. Perfusion compromise as a consequence of vasculitis in diabetes mellitus, tuberculosis, or the reduction of flow observed in the various causes of papillary necrosis sets the stage for ischaemic changes in the medullary pyramid.^5^

Lang EK et al,^6^ using the enhancement characteristics on the arterial corticomedullary phase, showed that in renal papillary necrosis the degree of enhancement was significantly lower than that of normal kidneys. In those patients who instituted therapy to relieve the underlying cause of papillary necrosis, at 3 months 57% of patients showed (20 out of 35) interval resolution of lesions.^6^ In this study, the stage of papillary necrosis is not evident, especially since CT cannot detect the ischaemic phase of papillary necrosis. Jung DC et al^5^ on CT urography described the CT findings in the early ischaemic phase of papillary necrosis as normal.

Thus, diffusion restriction, which occurs by virtue of ischaemia and cytotoxic oedema, when occurring at the tip of the medullary pyramid and calyx, is likely to represent the early ischaemic phase of papillary necrosis. This is similar to the pathophysiology of acute ischaemia and stroke causing diffusion restriction due to cytotoxic oedema in the brain.^7^ Diffusion restriction can occur in the brain because the white matter tracts introduce anisotropy. Studies by Fukuda M et al and Wu Y et al^8,9^ showed the anisotropic nature of diffusion in the kidney due to the radially oriented vessels and tubules.

However, based on the diffusion restriction at the calyx and the tip of the medullary pyramid, we propose that early medullary ischaemia can be detected, and thus treatment instituted, to reverse the inciting inflammation, vasospasm, vasculitis or compromise in microvascular perfusion, thereby preventing the progression of ischaemia beyond this stage.

Literature review showed only a case report by Schroeder J et al describing the features of renal papillary necrosis. In this case report they described features of papillary necrosis such as sloughed papilla in the calyx, clubbing of the calyx, a golf ball on a tee appearance and filling defects in the major calyx giving rise to a lobster claw appearance.^10^

In 1995, Rothpearl et al described MR urography for the purpose of imaging the renal system. They proposed that in obstructed systems the anatomy of the calyces and collecting system can be easily demonstrated. One of the patients in this case series had papillary necrosis. However, the findings of papillary necrosis seen in that patient were not described in the case series.^11^ In 1995, Hattery and King brought into question the efficacy of MR urography in making a diagnosis, given the resolution requirements needed to achieve good visualization of the calyceal and collecting system anatomy.^12^ Since then, until the case report published by Schroeder et al, to the best of our knowledge no study or case report has been published describing the findings of papillary necrosis on MR urography. Since then there has been tremendous progress in image acquisition techniques and hardware, enabling higher resolution of images.

In the index study, papillary necrosis has been diagnosed and findings described on MR urogram, performed on a 1.5 T MR scanner. Thus we propose that static MR urography is sufficient to establish a diagnosis of papillary necrosis.

Papillary necrosis can cause complications such as obstruction secondary to sloughing of papillae. The diffusion restriction at the calyces probably represents the finding of papillitis, that is, early ischaemic change at the tip of the medullary pyramid, which may later result in injury to the papilla, cause sloughing and later progress to intraluminal obstruction. Thus we propose that patients who have the finding of calyceal diffusion restriction, that is, ischaemia at the tip of the medullary pyramid, must be followed up more stringently so as to enable early intervention in the form of stenting or percutaneous nephrostomy if the patient develops features of obstruction. Further, in high-risk groups of patients prophylactic stenting may be advocated.

Thus, in view of the potential to change the course of management and prevent significant renal morbidity the finding of diffusion restriction in the tip of the medullary pyramid is a very important new finding. Further, this case series reiterates the fact that contrast administration may not be necessary for the diagnosis of papillary necrosis.

## Learning points

1. Diffusion restriction in the calyx and the tip of the renal pyramid is likely to be due to the ischaemic phase of papillary necrosis.
2. Findings of papillary necrosis seen on CT urogram and intravenous urogram (conventional) can also be seen on MR urogram without the administration of contrast.
3. With CT urogram and intravenous urogram the early diagnosis of papillary necrosis is difficult. However, with the use of MR urogram and diffusion-weighted imaging, early diagnosis of papillary necrosis is possible. This can permit risk stratification of patients, where high-risk patients may be offered early ureteric stenting to prevent the development of obstructive uropathy.

## Consent

The consent of the patients to publish the data and images was taken and is held on record.
